# Extemporaneous Formulations for Pediatric Patients: Global Necessities, Challenges and Opportunities

**DOI:** 10.3390/pharmaceutics18010126

**Published:** 2026-01-19

**Authors:** Vinita Balakrishna Pai, Milap Chand Nahata

**Affiliations:** 1 Division of Pharmacy Practice and Science, College of Pharmacy, The Ohio State University, Columbus, OH 43210, USA; 2 Institute of Therapeutic Innovations and Outcomes, Colleges of Pharmacy and Medicine, The Ohio State University, Columbus, OH 43210, USA

**Keywords:** extemporaneous formulations, pediatrics, compounding, personalized medicine

## Abstract

Many commercially available medications are often unapproved or unavailable in suitable dosage forms for specific patient populations, particularly infants and children. This necessitates the use of extemporaneously compounded formulations to deliver individualized doses based on body weight or body surface area, and when a medication is unavailable at an appropriate concentration or contains excipients potentially unsafe for certain patients. Extemporaneous compounding is required for oral liquids when patients are unable to swallow tablets or capsules. It is also needed for topical preparations and sterile dosage forms when commercial products are unavailable. Across regions, practices follow national pharmacopeial standards for both sterile and non-sterile compounding. Stability factors influencing the safety and efficacy of compounded formulations must be carefully considered when assigning appropriate beyond-use dates. While stability information is available for some medications in monographs, peer-reviewed literature, prescribing information, and investigator’s brochures, such data is often lacking for many compounded preparations. Emerging extemporaneous formulations—such as orodispersible films, nanoparticle systems, and 3D-printed compounds—offer potential advantages over traditional compounded formulations but present unique challenges to widespread implementation. Despite the justified clinical need for extemporaneous compounding, significant barriers remain, including limited access to medications, insufficient compounding expertise or resources, gaps in pharmacokinetic and safety data, and regulatory constraints. This review critically appraises the current state of extemporaneous compounding—drawing primarily on the United States of America frameworks—and highlights its continued necessity, associated challenges, and pragmatic solutions for advancing personalized pharmacotherapy across pediatric age groups worldwide.

## 1. Introduction

The practice of compounding a dosage form to address the medical needs of an individual patient has existed since at least the early 1600s, when practitioners known as apothecaries, primarily chemists, mixed and sold medicines [1]. The demand for compounding declined as over 90% of medicinal products used in patient care began to be commercially manufactured [2,3]. Nevertheless, extemporaneous compounding remains essential where authorized products do not meet patient needs, anchoring the two themes of this work: the necessities driving patient-specific therapy and the challenges that constrain safe, high-quality practice. Extemporaneous compounding remains a key component of pediatric pharmacy practice, providing patient-specific, sometimes life-saving medications to patients whose health care needs cannot be met by commercially manufactured dosage forms. In the United States of America (US), the compounding of extemporaneous formulations is currently more commonly performed within hospital pharmacies or health systems. Community pharmacies often have limited resources and expertise for compounding, with approximately 7500 of 56,000 pharmacies in the US providing extemporaneous compounding as a specialized function [4]. Many such pharmacies operate with dedicated facilities and quality systems, providing both sterile preparations and non-sterile dosage forms such as oral liquids, oral solid dosage forms, and topical formulations.

The United States Pharmacopeia (USP) Chapter <795> Pharmaceutical Compounding—Nonsterile Preparations (USP <795>) describes compounding as “the preparation, mixing, assembling, altering, packaging, and labeling of a drug, drug-delivery device, or device in accordance with a licensed practitioner’s prescription, medication order, or initiative based on the practitioner/patient/pharmacist/compounder relationship in the course of professional practice” [5].

According to the European Directive 2001/83/EC, compounding is classified as a magistral formula or an officinal formula. A magistral formula is any medicinal product prepared in a pharmacy according to a prescription for an individual patient. It is an extemporaneous preparation compounded for specific patients or patient groups and supplied immediately after preparation. The officinal formula is “any medicinal product which is prepared in a pharmacy in accordance with the prescriptions of a pharmacopoeia and is intended to be directly supplied to the patients served by the pharmacy” [6]. By contrast, stock preparations are compounded in advance and stored until a request for supply is received. On this basis, some European countries allow stock preparations alongside magistral or officinal formulas to ensure immediate availability at dispensing.

This article provides a comprehensive state-of-the art review of the role of extemporaneous compounding in current clinical practice involving pediatric patient care. It critically appraises the operational and regulatory challenges, including stability evidence and beyond-use dating, quality-system requirements, and regulatory heterogeneity, that influence safe implementation worldwide. We also highlight recent innovations, such as orodispersible films and tablets, nanoparticle formulations, and 3D-printed compounds. While these platforms can expand access to tailored therapies, they also introduce new validation, quality-control, and oversight requirements that must be addressed for routine clinical use. Finally, we outline pragmatic strategies and opportunities to improve quality, safety, and access, providing a concise framework for risk-based decision making by clinicians, pharmacists, and regulators. Relative to certain recent extensive review articles [7,8], the uniqueness of our critical review includes its broad scope, comprehensiveness, inclusion of the most recent studies and regulations, description of limitations of existing literature for application in patient care, and elaboration of opportunities as a roadmap for the future to advance the care of pediatric patients globally.

## 2. Literature Sources and Study Selection

For this narrative review, a literature search was conducted using the PubMed, Embase, Web of Science, and Scopus databases. Search terms included “extemporaneous”, “compounding”, “formulations”, “stability,” “pediatrics”, ‘prevalence”, “novel”, “3D-printing”, “orodispersible films”, “orodispersible tablets”, and various combinations thereof. Articles published in non-English languages, not relevant to pediatric patient care, and only available as Abstracts were excluded. Priority was given to review articles and studies that focused on any aspect of extemporaneous compounding or extemporaneous formulations. Additionally, the bibliographies of selected articles were examined to identify further relevant publications. Both authors agreed on the inclusion and exclusion of articles for performing this review. Although PubMed includes citations dating back to the 1960s, this review was restricted to literature published between January 2000 and May 2025 to ensure a focus on contemporary practices and recent developments in the field.

## 3. Clinical Need for Extemporaneous Compounding

Compounding oral extemporaneous formulations is often a necessity for pediatric patient populations, particularly those who cannot swallow solid dosage forms or for whom commercially manufactured liquid formulations do not meet the individualized dosing requirements.

Drug doses for pediatric patients are typically calculated based on body weight (mg/kg) or body surface area (mg/m^2^). This approach often results in specific doses that cannot be administered using commercially available formulations containing a fixed amount of the active pharmaceutical ingredient(s) (APIs) per tablet or capsule. In such cases, liquid dosage forms offer the advantage of enabling the administration of patient-specific doses. Many children under 6 years of age may be unable to swallow solid dosage forms, such as tablets or capsules, despite targeted training efforts [9]. Some infants may develop oral aversion and refuse to swallow food or medications, particularly if they have not utilized the oral route for an extended period. In such cases, the use of enteral feeding tubes—such as nasogastric (NG) or nasojejunal (NJ) tubes—may be required for both nutritional support and the administration of medications. It is essential that extemporaneously compounded formulations intended for these patients are appropriate for enteral tube delivery to ensure both the safety and efficacy of therapy. Oral aversion is not exclusive to the pediatric population; nearly 40% of adult patients may also experience oral aversion [10], particularly in relation to the administration of oral medications, thereby necessitating the use of NG or NJ tubes. In this context, compounded liquids should also consider viscosity/osmolality, material compatibility, and adsorption/occlusion risk to ensure reliable delivery via feeding tubes.

Certain drugs with a narrow therapeutic index, such as tacrolimus, require personalized therapy with careful dose titration to maintain serum concentrations within a defined therapeutic range, particularly in patients undergoing hematopoietic cell transplantation or solid organ transplantation. Achieving these individualized target concentrations may necessitate doses that are not commercially available, as tacrolimus is typically marketed only in capsule form. In such scenarios, the use of a liquid dosage formulation offers a practical solution, enabling precise dose adjustments to ensure effective and safe therapy. However, very low doses derived from concentrated stock solutions may increase measurement error; standardized dilutions and clear administration devices can mitigate this risk among pediatric patients.

In some cases, despite the availability of liquid dosage forms, compounding an extemporaneous formulation may be necessary because the dosage form contains excipients at concentrations that could be unsafe for the patient, especially premature newborns, neonates and infants. For example, dexamethasone intensol solution, 1 mg/mL for oral administration, contains a high concentration of alcohol (30% *v*/*v*), which may be harmful to premature newborns, term neonates, and infants. As a result, there is a need for extemporaneous compounding of a formulation that excludes alcohol to ensure safety and suitability for this vulnerable population. Extemporaneous compounding may be necessary if the concentration of the commercially available preparation is inappropriate for practical administration of a dose. Accurate dosing in pediatric patients can be difficult when commercially available formulations contain high concentrations of API(s) intended for older populations, as the measurement of extremely small volumes for the doses required may lead to administration errors. For example, furosemide injection is commercially available in a 10 mg/mL concentration [11]. A 1 mg/kg dose of furosemide for a 750 g baby would be 0.75 mg or 0.075 mL of the undiluted medication, which would be difficult to prepare accurately without further dilution. Beyond excipient ethanol, other excipients (e.g., propylene glycol, sorbitol, benzoates) may warrant avoidance or tighter limits in neonates and young infants.

The treatment of rare diseases often relies on medications classified as orphan drugs, such as L-carnitine and L-arginine, which may not have commercially available formulations meeting the unique requirements of all patient populations [12]. Due to limited demand, pharmaceutical companies are often unwilling to invest in manufacturing appropriate dosage forms for these drugs, making extemporaneous compounding essential to meet individual patient needs. Sometimes specific combinations of APIs may be essential for effectively treating various dermatological conditions. However, these tailored formulations are frequently unavailable commercially, necessitating dermatologists to prescribe individualized combinations for compounding [13,14,15]. Extemporaneously compounded medications play a crucial role in the management of acute or chronic pain, as well as in the alleviation of symptoms during palliative and hospice care. In many cases, the specific therapeutic needs of patients may not be adequately addressed using commercially available products, making compounding an essential practice in these settings [16,17,18].

Shortages of commercially available dosage forms, particularly those used to address specific patient needs in acute care settings, have significantly contributed to a resurgence in extemporaneous compounding. Notable examples in the US include the shortages of ibuprofen suspension in 2023 and amoxicillin and oseltamivir suspensions in 2022 [19,20]. These shortages have been exacerbated by supply chain disruptions resulting from epidemics, pandemics, and natural disasters. As of March 2025, the American Society of Health-System Pharmacists (ASHP) reported 270 active drug shortages nationwide [21]. Collectively, these scenarios illustrate the clear clinical necessity for extemporaneous compounding but also highlight practical challenges—accurate low-dose measurement, tube-administration compatibility, excipient safety in vulnerable populations, and the need for justified beyond-use dates—that must be addressed to ensure safe, effective patient care.

### 3.1. Critical Review of Global Extemporaneous Compounding Practices in Pediatric Patient Care

Many drugs lack pediatric labeling and child-friendly formulations because research has traditionally focused on adults. To address this, the FDA’s Best Pharmaceuticals for Children Act (BPCA) and Pediatric Research Equity Act (PREA), along with the European Medicines Agency (EMA), have established regulations to promote pediatric drug development [22,23,24]. Despite these efforts to increase pediatric labeling for newer drugs, many older, off-patent essential medicines—such as those on the WHO Model List of Essential Medicines for Children (EMLc) still lack appropriate pediatric dosage forms [25]. A review analyzing studies published between 2012 and 2022 across Europe, North America, Southeast Asia, Australia, and Africa found that off-label drug use remains prevalent in patients aged 0–18 years, with rates ranging from 3.3% of over 4 million prescriptions in Italy to 71.5% of nearly 2000 prescriptions in Indonesia [7].

Given the widespread global use of off-label medications in pediatric populations, we conducted a focused literature review (2000–2025) to assess the prevalence of extemporaneous compounding and identify the most commonly compounded formulations in children. Most of the 17 included studies used cross-sectional surveys and observational designs; a few employed prospective approaches, such as nested case–control or pilot studies. These studies examined compounding practices, prevalence, and perceptions across healthcare settings in Europe, North America, Oceania, Asia, and Africa. Pharmacists, particularly those working in hospitals, communities, and independent settings, were the primary respondents. Some studies also surveyed physicians and nurses, especially in pediatric or hospital-based contexts. Five studies focused specifically on pediatric populations, targeting pediatric hospitals, formulations, or drug manipulation practices. Survey response rates ranged from 27.3% to 98%. Compounding was routinely performed across settings, with pharmacies averaging 12.5 compounded preparations per day and 3–12% of prescriptions requiring compounding. About 94% of pharmacists reported providing compounding services, and 20% of physicians prescribed medications requiring compounding. In pediatric inpatient settings, 28% of children received at least one extemporaneous liquid formulation. Frequent manipulation of oral dosage forms (e.g., splitting/crushing tablets) was consistently reported.

The findings indicated that non-sterile extemporaneous compounding is a routine practice in pediatric care worldwide. Oral liquid formulations and topical dermatologic preparations were the most frequently compounded dosage forms, followed by capsules, powder papers, and manipulated solid dosage forms such as tablets that were crushed or split. (Table 1). Commonly compounded drugs included spironolactone, furosemide, omeprazole, lansoprazole, captopril, sildenafil, hydrochlorothiazide, caffeine citrate, and prednisone/prednisolone. A 2024 global survey completed by 479 WHO regions found that oral liquids were the most frequently compounded preparations, with 90% of the participants doing so, and the diuretics, drugs for acid-related disorders, and beta-blockers were the top three compounded classes for the pediatric patients [26].

The reviewed studies offer valuable insights into extemporaneous compounding practices across diverse global settings. However, several methodological and contextual limitations impact the generalizability and comparability of the findings. Study designs were highly heterogeneous, ranging from surveys to observational assessments, with wide variation in the metrics and response rates used to evaluate compounding prevalence. Some studies reported the percentage of prescriptions compounded, while others quantified the volume, frequency, or number of manipulated dosage forms. The duration of data collection also varied from daily to monthly assessments. Conducted across multiple continents over an extended period, the studies reflect regional differences in regulatory frameworks, drug formularies, healthcare infrastructure, and access to commercially available pediatric formulations. Additionally, variation in study respondents, including pharmacists, physicians, and nurses, further contributes to the heterogeneity of the data. Taken together, these patterns underscore a clear clinical need for extemporaneous compounding but also pose persistent challenges for evidence synthesis—namely, heterogeneous metrics, variable time horizons, and region-specific regulatory contexts—that should be considered when interpreting comparative prevalence estimates.

### 3.2. Essential Components of Extemporaneous Compounding

#### 3.2.1. Information Resources for Compounding

Extemporaneous compounding at the point of care is feasible only when the compounding facility possesses an established infrastructure that complies with applicable national regulations. For instance, in the US, USP Chapter <797> on Pharmaceutical Compounding—Nonsterile Preparations provides guidelines for the compounding of nonsterile products in accordance with established good practice standards.

Information on the formulation of an extemporaneous preparation—including ingredients, compounding methods, storage containers, optimal storage conditions, and reliable stability data may be obtained from various sources. The most accessible resource in a hospital setting is typically the country’s pharmacopoeia and national formularies, provided they include official monographs for compounded preparations that encompass all relevant information. The USP-National Formulary (NF) currently has over 200 such monographs [45]. For drugs with approved pediatric labeling, relevant information may be found in the package inserts for brand-name products. Additionally, the investigator’s brochure for drugs under study, particularly those focused on pediatric applications, may provide details on dose preparation or manipulation. However, obtaining this proprietary information can be challenging. Furthermore, the investigator’s brochure content is trial-specific and not a substitute for validated compounding data; it should be used only if appropriate compatibility/stability and, where relevant, bioequivalence have been demonstrated. Tertiary references such as the Pediatric and Neonatal Dosage Handbook, Pediatric Drug Formulations, and Extemporaneous Formulations for Pediatric, Geriatric, and Special Needs Patients have compiled the necessary published information for compounding extemporaneous formulations and are periodically updated [46,47,48]. If these resources do not yield the required formulation information, a search of the primary literature should be conducted. Access to national pharmacopeias, formularies, and tertiary references may be limited due to cost. However, in such situations, freely available extemporaneous formulation recipes published by hospital pharmacies online may serve as alternatives (Table 2). Before use, it is essential to evaluate the accompanying stability data and ensure that the preparation meets national standards for non-sterile compounding.

If all attempts to acquire the necessary information fail, the drug can still be administered to the patient; however, this may necessitate manipulation and administration of the dose at the bedside for immediate administration. This process carries a significant risk of errors, particularly if the dose for an infant or young child represents a fraction of the commercially available dosage form. Additional complications may arise if the drug requiring bedside manipulation is hazardous, necessitating specific precautions while handling it.

#### 3.2.2. Non-Sterile Extemporaneous Formulations

1. Liquid Dosage Formulations for Personalized Patient Care

A comprehensive review of safe and effective extemporaneous formulation can be found in the most current USP or the technical bulletins published by ASHP [49,50]. These may also be available in the national pharmacopoeias of individual countries or published by their respective pharmacy organizations. Liquid formulations are the most frequently compounded for use in children [26]. Because many APIs in the commercially available solid dosage forms used in extemporaneous compounding of liquids are insoluble in water, suspensions are among the most commonly compounded formulations. The base vehicle for compounding suspensions typically consists of a suspending agent and a sweetening agent (e.g., syrup) in a 1:1 proportion. Numerous commercially made ready-to-use suspending agents, such as Ora-Plus^®^, and sweetening agents such as Ora-Sweet^®^, and others, including Ora-Blend Flavored^®^, SuspendRx^®^, and SyrSpend^®^, are available. Some of the sweetening agents are available in sugar-free form. However, these products may be cost-prohibitive or unavailable in some countries. A stable bedaquiline suspension was made using widely available cane sugar for preparing simple syrup and “Thick & Easy” food starch as a thickening agent for pediatric patients in Africa [51]. If a ready-to-use suspending agent is unavailable, an extemporaneous preparation may be made with regionally available ingredients—for example, a 1:1 mixture of 1% methylcellulose suspension and Simple Syrup USP. Other examples of extemporaneously compounded suspending agents may be found in each country’s pharmacopoeia or national formulary. In the USP, examples include Vehicle for Oral Suspension, Suspension Structured Vehicle, and Sugar-Free Suspension Structured Vehicle USP [45].

The acceptance of extemporaneously compounded formulations is essential for ensuring patient adherence to medication regimens, particularly in pediatric patients. As noted, “a spoonful of sugar helps the medicine go down,” which highlights the importance of palatability in treatment adherence. Organoleptic properties, including sweetness, taste, odor, texture, color, and flavor, play a critical role in the acceptance of these formulations. For patients with dietary carbohydrate restrictions, such as those with diabetes mellitus or following ketogenic diets, sugar-free sweetening agents provide suitable alternatives. A specific flavoring agent can be strategically added to mask undesirable tastes associated with certain APIs; for example, chocolate can effectively disguise bitterness [52]. Chocolate Syrup USP may be used both as a sweetening and flavoring agent [45]. Other examples of extemporaneously compounded flavoring agents that can also serve as sweetening agents may be found in each country’s pharmacopoeia or national formulary. In the USP, examples include Cherry Syrup, Orange Syrup, Peppermint spirit, and Vanilla tincture [45]. Additionally, flavors can be paired with coloring agents to enhance aesthetics. However, their addition should be approached with caution, especially if they were not included in the original formulation with stability data, as it may compromise the formulation’s stability and safety. Changes in temperature, pH, light exposure, and the presence of oxidizing or reducing substances can adversely affect added colors. In cases where stability data for flavoring or coloring agents are lacking, these agents may be added to individual doses as needed immediately before administration. Availability and affordability vary by country; where products are not available, in-house vehicles must be prepared and documented according to local standards.

To stabilize and preserve, certain formulations may require the addition of acids, bases, pH buffering agents, or solubilizing agents such as propylene glycol, ethanol, and glycerol, as well as preservatives like parabens and benzyl alcohol. However, caution is necessary when using these additives, as the original dosage form intended for extemporaneous compounding may already contain one or more of them, increasing the total exposure and risk of toxicity—particularly in premature newborns, neonates, and infants, thereby raising the potential for adverse effects (Table 3) [53,54]. Under such circumstances, additives should be used in minimal amounts or not at all, while still meeting the stability, palatability, and safety criteria for the dosage form.

Using extemporaneously compounded liquid dosage forms for pediatric patients may not always be feasible, particularly when the beyond-use-date (BUD) is very short, necessitating frequent visits to the pharmacy for prescription refills. This can prove impractical due to work obligations, distance from the pharmacy, or lack of transportation. In such cases, manufactured tablets may be cut or split, and capsules may be opened with their contents mixed with food items such as applesauce, pudding, or ice cream, or liquids like water, juice, or formula. However, these methods may compromise the physical and chemical stability of the medication, particularly if the original formulation is extended-release, delayed-release, or enteric-coated, which could lead to dosing errors from inaccurate splitting, spillage, or loss of specific release technology during reconstitution. Additionally, mixing with large quantities of food should be avoided, as patients may not consume the entire portion, resulting in inadequate dosing. For water-soluble APIs, crushed tablets or capsule contents can be mixed with a desired volume of water to achieve a precise dose based on mg/mL; however, this process carries the risk of preparation and calculation errors. Any manipulation should be supported by clear instructions, appropriate measuring devices, and, where feasible, compatibility/stability information to minimize pharmacokinetic and pharmacodynamic variability.

2. Novel Extemporaneous Solid Dosage Formulations for Personalized Patient Care

Assuming the patient’s ability to swallow is unimpaired, commonly compounded solid oral dosage forms include powder papers (sachets), capsules, and tablets, all of which are resource- and labor-intensive to prepare, even with access to capsule-filling and tablet-compression equipment.

Novel dosage forms—such as orally disintegrating tablets, fast-dissolving tablets, orodispersible films, lozenges, hard candy, and troches are classified as oral transmucosal drug delivery systems [55,56,57]. They facilitate systemic administration of the API, particularly in patients who are unable to swallow traditional solid or liquid dosage forms or who require rapid drug release and a prompt clinical response. Drug release from these dosage forms occurs primarily through dissolution in saliva, followed by absorption across the oral mucosa, which can bypass first-pass hepatic metabolism and enhance bioavailability. The selected excipients must facilitate the disintegration and release of the API within 10–15 min or less, to ensure timely mucosal absorption and therapeutic effects. Given their prolonged contact with the oral mucosa, these dosage forms must also possess acceptable taste, flavor, and texture. Their formulation and use should be supported by documented stability data.

Orally dissolving films and orally disintegrating tablets may offer the advantages of ease of medicine administration, rapid onset of action, increased bioavailability, and improved adherence [56]. Buprenorphine, ondansetron, levocetirizine, rizatriptan, clobazam, diazepam, nicotine, sildenafil, tadalafil, dextromethorphan and diphenhydramine are among the commercially available medicines approved by the FDA as films. Loratadine, cetirizine, ondansetron, and tramadol are examples of orally disintegrating tablets approved by the FDA. These technologies are rarely used for preparing extemporaneous formulations for many reasons stated above.

The use of three-dimensional (3D) printing technology in extemporaneous compounding is an emerging and promising approach that significantly advances the practice of personalized medicine [58,59]. This flexible platform enables the digital design and fabrication of solid dosage forms using specialized software and computer-aided design (CAD) tools, which define the geometry and dimensions of the final product. In this process, a 3D-printer is loaded with a mixture of APIs and excipients and constructs the dosage form layer by layer. The printing parameters—such as resolution, temperature, and speed—are programmed based on the physicochemical properties of the drug, the type of printer used, and the desired characteristics of the final product. Among the various 3D-printing technologies, stereolithography (SLA) is particularly notable for its high resolution and precision, with the ability to achieve fine structural details. 3D-printing has demonstrated the potential to individualize drug delivery systems by tailoring the release profile to the patient’s specific pharmacokinetic data. The 3D approach enables the development of complex dosage forms such as polypills, which incorporate multiple APIs into a single unit, thereby improving patient convenience and adherence. Furthermore, 3D-printing facilitates the production of tablets in various shapes, sizes, colors, and flavors, which can enhance patient acceptability, especially in pediatric populations. Unlike traditional pharmaceutical manufacturing, 3D-printing supports on-demand, point-of-care production of personalized medications, representing a transformative advance in extemporaneous compounding and precision pharmacotherapy. Current advancements in 3D-printing of pharmaceuticals are focused on the development of specialized printers, printing software, and suitable pharmaceutical excipients that enable the formulation of dosage forms with predictable drug release profiles and acceptable stability with assurance of quality and reproducibility. In parallel, evidence of economic feasibility (cost-effectiveness/scalability), regulatory frameworks, assurance of drug excipient compatibility in the printing process, potential occupational exposure risks for pharmacy staff, and potential impact of this technology on patient care and safety must be established before this technology can be widely adopted in the pharmaceutical industry and clinical practice. It is not surprising that levetiracetam is the only commercially available 3D-printed medicine approved by the FDA, and this technology has not been used much for preparing extemporaneous formulations among patients in the US, largely due to regulatory, technical, and cost barriers.

Medicines in nanoparticles can be delivered to targeted areas (e.g., pathogens or cancer cells) while minimizing harm to healthy cells, improving bioavailability and releasing API(s) gradually for sustained therapeutic effectiveness [60]. Examples of commercially available medicines in the US are liposomal amphotericin B, doxorubicin, daunorubicin, cytarabine and vincristine, PEGylated nanoparticles in pegfilgrastim, and crystalline nanoparticles in Elixophyllin^®^. However, this technology is largely used in industrial manufacturing and rarely employed in preparing extemporaneous formulations due to many limitations similar to 3D-printed medicines.

### 3.3. Factors Influencing Utilization of Extemporaneous Formulations in Clinical Practice

The efficacy and safety of compounded formulations depend on their ability to remain stable throughout clinical use. Therapeutic stability ensures that the drug’s therapeutic effect remains unchanged until the BUD, the date beyond which the physical, chemical, microbiological, therapeutic, and toxicological stability of the compounded formulation cannot be guaranteed [5]. This date is determined from the time the formulation is compounded and cannot be directly extrapolated from the expiration date of the commercial dosage form used in the compounding process. As a general expectation, formulations with storage parameters and BUDs that maintain the potency of active ingredients within 90 to 110% of the initial concentration, along with assurance of physical, chemical, and microbiological stability, are recommended. Bioavailability, pharmacokinetic, efficacy, safety and outcomes studies are rarely available for extemporaneously prepared formulations.

When a BUD for a formulation that meets physical, chemical, microbiological, and therapeutic stability is unavailable, the BUD recommendations provided in the pharmacopoeia applicable to the respective country or USP <795> for non-sterile preparations should be utilized. These BUDs are applicable in the absence of stability information for specific preparations through a USP-NF monograph or published stability literature specific to the formulation. The BUD must not exceed the expiration date of any components used in the compounding process. In the revised USP <795>, the determination of BUDs for non-sterile oral liquid formulations is based on “water activity” (“a_w”) (Table 4), which is critical in determining the susceptibility of a non-sterile preparation to microbial growth [40]. Additionally, it also informs the potential for degradation of the active ingredient due to chemical reactions that may be induced by the presence of water. The update places greater emphasis on microbial risk (as reflected by “a_w”) and, where applicable, on the performance of the preservative system; default BUDs may be extended only when supported by appropriate, stability-indicating data.

These BUDs should be applied with caution in certain cases. For example, captopril (0.75 mg/mL) in Cherry Syrup remains stable for only 2 days at room temperature or when refrigerated. It is stable for ≤10 days in a 1:1 mixture of Ora-Sweet^®^ and Ora-Plus^®^ or Ora-Sweet^®^ SF and Ora-Plus^®^, depending on the storage temperature [61]. Therefore, without specific stability data, labeling these captopril formulations with a BUD of 14 days in accordance with USP guidelines would be inappropriate. The 2023 revised USP addresses this concern by recommending a shorter BUD if the physical and chemical stability of the compounded formulation is less than the USP-recommended BUD (Table 4). In practice, compounders should default to the shorter of: (i) the USP default BUD based on dosage form and “a_w”, or (ii) the BUD supported by formulation-specific stability data. Further, it is crucial to ensure that the BUDs were determined from well-designed studies utilizing validated methodologies, analyses and interpretations of the data for application in patient care.

### 3.4. Challenges and Safety Concerns with Extemporaneous Compounding

#### 3.4.1. Clinical Challenges in Assigning BUDs for Compounded Non-Sterile Preparations

With revised BUDs per USP <795>, any extemporaneously compounded oral liquid dosage form with an “a_w” of ≥0.6, devoid of a preservative, is assigned a BUD of 14 days in the absence of a USP-NF monograph or specific stability data for compounded non-sterile preparations [49]. This raises the question of whether the BUDs recommended in published stability studies for non-preserved aqueous oral dosage forms remain valid if such formulations contain no preservatives. Adding a preservative to an existing formulation with established stability data to extend the BUD beyond 14 days could potentially compromise its stability. Consequently, these updates necessitate repeating or supplementing published stability studies with the inclusion of preservatives to ensure that the API concentration remains within 90–110% of its original level for the recommended BUDs. However, preserved aqueous formulations are generally limited to a BUD of 35 days when stored under controlled conditions or refrigerated, regardless of how long the API remains within 90–110% of its original concentration. The BUD may be extended—up to the time point at which the API remains within 90–110% of its initial concentration or 180 days, whichever is shorter—only if the formulation passes antimicrobial effectiveness testing in accordance with USP <51> [62]. The revised USP <795> permits the use of bracketing in stability studies, employing validated analytical methods and antimicrobial effectiveness testing at both the lowest and highest concentrations of the API. If both concentrations meet the required criteria, the resulting stability data may be extrapolated to support BUDs for all intermediate strengths within that range. Adhering to the revised BUDs for oral liquid formulations may necessitate substantial workflow adjustments for pharmacists and pharmacy technicians in healthcare system pharmacies. Shorter default BUDs and preservative requirements will require more frequent compounding, increasing demands on time and resources. Additionally, significant investment will be needed to reformulate many existing extemporaneous preparations with the addition of preservatives, despite previously demonstrated API stability under recommended storage conditions. Physical, chemical, therapeutic, and microbiological stability testing will also need to be repeated to comply with the updated requirements.

#### 3.4.2. Regulatory Oversight and Safety of Compounded vs. Commercially Manufactured Drug Products

Drug regulatory agencies require preclinical and clinical studies to demonstrate the safety and efficacy of dosage forms prior to approval. Commercial manufacturing of approved formulations is regulated by national laws enforcing current good manufacturing practices (cGMP). In the US, cGMP regulations under the Federal Food, Drug, and Cosmetic Act (FD&C Act) set minimum standards for manufacturing, packaging, and marketing [63]. The WHO also issues cGMP guidelines, adopted into national laws by many countries [64]. In contrast, compounding pharmacies in most countries, including the US, are not subject to centralized regulatory oversight by agencies that govern pharmaceutical manufacturing, nor are they required to comply with cGMP. In the US, pharmacy practice, including compounding, is regulated by state boards of pharmacy. Most states require adherence to USP Chapter <795> for non-sterile compounding and Chapter <797> for sterile compounding. Compounded drugs are not held to the same safety, potency, or efficacy standards as those enforced by cGMP for commercially available products. Commercial drugs must include national FDA-approved labeling, whereas compounded drugs are subject only to state-level labeling rules. Adverse event reporting and FDA advertising regulations also do not apply to compounding pharmacies.

Extemporaneously compounded formulations have been associated with significant safety concerns. A 2001 FDA survey found that one-third of compounded products failed potency testing, compared to just 2% of the commercial drugs [65]. A 2006 follow-up revealed no improvement, with potencies ranging from 68% to 268%. Inconsistent procedures were implicated as likely contributors to safety issues. From 2001 to 2019, 1562 adverse events linked to compounding errors were reported in the US, including 116 deaths [66]. Common causes included contamination of sterile products, excipients of unknown grade, incorrect potencies, and mislabeling of ingredients. The lack of cGMP compliance and limited oversight were identified as key factors contributing to these incidents.

Sterile product compounding presents specific safety concerns. It remains uncertain whether USP <797> standards ensure sterility to the same degree as terminally sterilized, cGMP-manufactured products. Multiple outbreaks linked to bacteremia, blindness, and fungal meningitis have been traced to contaminated sterile preparations [67,68]. The 2012 fungal meningitis outbreak from preservative-free methylprednisolone resulted in 793 infections and 76 deaths, highlighting the dangers of large-scale compounding under lax oversight [69]. The growth of home infusion services and the outsourcing of parenteral products has expanded extemporaneous compounding beyond hospital pharmacies, leading to the creation of large-scale compounding facilities. These facilities resemble drug manufacturers but operate under pharmacy regulations. This regulatory gap contributed to the 2012 fungal meningitis outbreak [69].

In response, the 2013 Drug Quality and Security Act created Sections 503A and 503B of the FD&C Act [70]. Section 503A governs patient-specific compounding, regulated by state boards and USP standards. These pharmacies are not required to comply with cGMP but must conduct environmental monitoring and may establish expiration dates using internal or published stability data. Section 503B covers FDA-registered outsourcing facilities engaged in bulk compounding, which must comply with cGMP, register with the FDA and Drug Enforcement Agency, and undergo risk-based inspections. The US FDA maintains an active website that provides compounding risk alerts to inform health care professionals and compounders about risks associated with compounded drugs, including adverse events, outbreaks, and drug quality concerns. These alerts are intended to help practitioners more effectively protect patients from unsafe, ineffective, or poor-quality compounded medications [71]. Canada adopted a similar approach in 2009, with the Health Products and Food Branch Inspectorate distinguishing between compounding and manufacturing [72]. These adverse events have increased the legal liability of prescribers and compounding pharmacists. The FDA discourages compounding when approved alternatives exist, and professional societies now recommend informed consent when using compounded drugs as alternatives to commercially available dosage forms.

#### 3.4.3. US and EU Regulatory Responses to Gaps in Pediatric Labeling and Formulations

The US legislative initiatives, the BPCA and PREA, encouraged pediatric drug development and labeling. Between 2002 and 2019, 768 products received pediatric labeling changes, 63% under PREA, 21% under BPCA, and 16% under both, demonstrating the stronger influence of PREA’s mandatory approach compared to BPCA’s voluntary incentive [73]. The EU’s 2007 Paediatric Regulation led to significant advances in pediatric drug development, with over 260 child-specific authorizations by 2016 and more than 1000 pediatric investigation plans (PIPs) initiated [74]. Although 60% of PIPs were completed by 2019, only 55% qualified for extended market protection. These extensions delay competition and increase industry revenue, often at added cost to consumers and insurers.

Despite regulatory progress, only one-third of the drugs on the WHO EMLc have age-appropriate formulations, particularly for children aged 1–5 years. This gap persists largely because the EMLc primarily includes older or off-patent medications, which are not subject to the mandates or incentives provided under BPCA, PREA, or the EU Paediatric Regulation.

#### 3.4.4. Standardizing Extemporaneous Formulation Concentrations to Improve Efficiency and Reduce Errors

Medication errors causing grievous harm can occur with extemporaneously compounded formulations even when stability data are available. The lack of standardized concentrations for extemporaneously compounded formulations poses a significant risk of medication errors, particularly for those formulations that have varying stability and BUDs for different concentrations. Patients and caregivers typically report drug doses in milliliters when the dosage form is a suspension or solution. However, when a drug has multiple extemporaneous formulations with different concentrations, failing to confirm the concentration being used by the patient can lead to dosing errors. Documenting the dose in milliliters without inquiring about the specific concentration may result in incorrect dosing. A medication error involving extemporaneous formulations is likely to occur when prescriptions are transferred between different settings (e.g., from inpatient to outpatient or from one healthcare institution to another) if the concentration of the compounded formulation varies at any step in the transition and the dose is not adjusted accordingly based on the concentration.

Standardization of these formulations could substantially reduce the incidence of medication errors during compounding, dispensing, or administration. The American Society of Health-System Pharmacists (ASHP) has recommended standardized concentrations for intravenous and oral liquid dosage forms for use across all healthcare settings [75]. This ASHP “Standardize 4 Safety Initiative” provides a list of standardized concentrations for intravenous medications and compounded oral liquids for both adult and pediatric patients. Healthcare systems are encouraged to adopt these standard concentrations to minimize medication errors in patient care.

#### 3.4.5. Affordability of Extemporaneously Compounded Preparations

The costs of compounding, testing, and storing extemporaneous formulations pose significant challenges. Health systems operating 503A pharmacies, especially those with high compounding volume, may benefit from investing in in-house stability testing facilities. Reimbursement could help offset these expenses, but in the US, obtaining reimbursement can be difficult. Insurers may require time-consuming prior authorizations, and payment may not fully cover costs, particularly when using high-cost injectable ingredients. Many compounding pharmacies bill patients directly, increasing their financial burden.

In contrast, countries with nationally funded healthcare systems (e.g., much of Europe) are less affected by reimbursement limitations, while in nations without insurance coverage, such as parts of Africa and Asia, costs are typically paid out of pocket. Where demand is high, outsourcing to 503B facilities may offer a practical solution not only for patient-specific needs but also for supplying health systems more broadly. Further, economic modeling has been rarely done to establish the cost–benefit or cost-effectiveness of extemporaneous formulations.

## 4. Future Roadmap of Opportunities and Conclusions

The practice of extemporaneous compounding is essential in addressing the critical gap in medication availability for vulnerable pediatric populations, such as infants and children, who often require tailored or off-label dosage forms. Each country should identify the highest priorities based on its unique needs and resources to meet the needs of pediatric populations. Emerging extemporaneous solid dosage forms such as orodispersible films and 3D-printed compounds hold great promise for advancing personalized pharmacotherapy. However, their widespread use remains limited by technical, regulatory, quality control and cost constraints. While the necessity for individualized formulations cannot be overstated, the challenges associated with compounding practices, ranging from resource and expertise limitations to regulatory and reimbursement hurdles, pose significant barriers to effective implementation. In addition, default BUD frameworks and heterogeneous evidence for stability, preservative efficacy, and bioavailability introduce operational complexity that must be actively managed. Where feasible, standardizing concentration and documenting compatibility for enteral administration can further reduce error and variability. It is imperative for all stakeholders, including healthcare professionals, payors, and regulatory bodies, to collaborate on developing harmonized, risk-based guidelines and providing resources that enhance the safety, effectiveness, and accessibility of compounded medications in diverse health-system contexts. Global priorities include the needs for: (i) well-deigned and validated stability-indicating studies aligned with “a_w” and, where applicable, antimicrobial effectiveness testing; (ii) strengthened quality systems and control, training, and capacity-building (especially in low- and middle-income settings); (iii) documentation of outcomes studies of effectiveness, safety and patient- and caregiver acceptance and adherence of the formulations among patients when feasible; (iv) shared, open access formularies or repositories of validated formulations and standard concentrations adopted by hospital networks/consortia; (v) regulatory harmonization among countries; (vi) increased funding of research to improve the access and use of effective and safe extemporaneous formulations for infants and children; and (vii) reimbursement and procurement models that recognize the true cost of safe compounding in both publicly funded and out-of-pocket payment systems. By doing so, we can optimize therapeutic outcomes and ensure that all pediatric patients—regardless of region—receive the individualized care they deserve.

## Figures and Tables

**Table 1:** Curated Overview of Prevalence of Non-sterile Extemporaneous Compounding Practices Across Countries.

Study Design/Reference	Geographic Locations	Compounded Prescriptions (Rx’s) or Reported Use	Age Group	Most Compounded (c) or Manipulated (m) Dosage Form	Top 5 Drugs Frequently Compounded in Extemporaneous Formulations
Questionnaires answered by hospital pharmacists from 18 European countries to determine the most frequently extemporaneously compounded oral dosage forms, including the drug names, total volumes, and number of times the dosage forms were made in a 6-month period. Brion F et al. 2003 [27].	Belgium, Croatia, Denmark, England, Finland, France, Germany, Ireland, Italy, Norway, Scotland, Slovenia, Spain, Sweden, Switzerland	21/41 (51%) questionnaires returned. Over a six-month period: morphine was compounded 447 times, totaling 76,170 mL; isosorbide, totaling 4028 number of powder papers; hydrochlorothiazide, totaling 14,060 capsules	Pediatric (specific age distribution not provided)	Oral liquids >60% (c) in Denmark, England, Ireland, Norway, Sweden	Morphine, prednisolone, trimethoprim, captopril, midazolam
				Paper powders (c) in Finland, Italy, Scotland	Isosorbide, glycine, caffeine citrate, L-citrulline, vitamin E
				Capsules (c) in Belgium, Croatia, France	Hydrochlorothiazide, spironolactone, captopril, prednisolone, folic acid
Prospective, nested, case–control study of pharmacies (*n* = 79) dispensing Rx’s for compounded medicines during one predetermined study day. Buurma H et al. 2003 [28].	The Netherlands	3.4% (991/28,711) Rx’s/day were for compounded medicines 12.5 compounded medicines per pharmacy per day (mean)	Adults and pediatric patients (<12 years of age) more likely to receive Rx for compounding	Dermatological (62.1%) (c) Oral solutions (7.4%) (c) Ear, nose, throat products (7.1%) (c)	Specific drug names not provided Drugs affecting central nervous, cardiovascular, and gastrointestinal systems
Prospective, survey-based, observational study to determine the extent of Rx compounding in independent community pharmacies. McPherson et al. 2006 [29]; Martin KS et al. 2009 [30].	US (Illinois, Missouri, Kansas, Iowa)	22.92% (370/1610) response rate, 347 provided compounding; 333 analyzable 2.3% (7768/344,677) Rx’s compounded/week ≥5% of the total Rx’s compounded by 12.3% of the 347 compounders	Not specified	Dermatological (solutions, ointments, creams, gels) (90.7%) (c) Oral solutions (73.2%) (c) Oral suspensions (70.4%) (c) Dermatological and oral capsules are compounded at least once per week by 30% to 46% of the independent pharmacies	Not provided
Retrospective review of onsite logbooks to determine the extent and nature of extemporaneous compounding of liquid preparations in New Zealand. Kairuz T et al. 2007 [31].	New Zealand	2015 total products compounded over a 7-month period (in 2004); 252 per month (mean)	Pediatric patients (neonates, infants and children) included; inclusion of adult patients not specified	Oral dosage forms (152) (c) Topical dosage form (100) (c) Oral suspension is the most common oral dosage form compounded	Omeprazole, sotalol, labetalol, diazoxide, and clonidine
Prospective survey of children’s hospitals (*n* = 20) to understand the scope, frequency, and use of extemporaneous liquid formulations. Lugo, R et al. 2009 [32].	US	28% of the median of 8400 inpatient admissions per hospital over a 12-month period received at least one extemporaneous liquid formulation	Pediatric (newborn to 18 years)	Liquid (c)	Lansoprazole, spironolactone, captopril, sildenafil, ursodiol
Cross-sectional descriptive survey of community pharmacists (stage 1) and physicians (stage 2) to determine the extent of prescription compounding by community pharmacists in the West Bank. Zaid, A et al. 2012 [33].	Cities and villages in the 11 districts of the West Bank	72.2% (153/212) of pharmacies provided compounding services. 1.55% (1973/126,840) of total Rx’s were compounded 20.7% (37/179) of physicians prescribed a medication that required compounding and prescribed less than 20 Rx’s per month	Not specified	Topical preparations (97.3%) (c) Oral solutions (78.4%) (c) Oral suspensions (43.2%) (c)	Not provided
Observational study (over 2 weeks) assessing the extent of drug manipulations conducted in clinical practice in inpatient settings. Questionnaire survey of experiences and views of pediatric nurses about these manipulations. Richey RH et al. 2013 [34].	Britain	Total 310 manipulations of drugs/dosage forms identified	Pediatric (2 to 19 years of age)	Tablets (61.6%) (m) Intravenous injection (21%) (m) Sachet (9.7%) (m) Transdermal patch (3.2%) (m)	Analgesic, proton pump inhibitor, antimuscarinic agent, antiemetic, alginate preparation
		258 potential drug manipulations reported in 27.3% (153/560) of questionnaires returned; 188 (73%) of these were valid manipulations			
Pilot study to determine the extent of compounding by reviewing batch records over a period of 24 months. Masupye IM et al. 2015 [35].	South Africa	27.6% preparations compounded per month (691 batch records assessed)	Not specified	Solutions 43.9% (c) Creams 33% (c) Ointments 13.6% (c)	Betamethasone, other corticosteroids
Prospective cross-sectional study at three hospitals to assess the extent of manipulation of oral medicines, the type of and dosage form manipulated over a four-week period. Bjerknes K et al. 2016 [36].	Norway	17% (509/3070) of oral administrations were manipulated	Pediatric (newborn to 17 years of age) Number of manipulations by age: 28 days to 23 months—22.5% (175/777) 6–11 years—28.8% (276/957)	Tablets (87%) (m) Capsules (11%) (m)	Prednisolone, metronidazole, ketobemidone hydrochloride, nitrazepam, azathioprine
Observational study with cross-sectional descriptive survey conducted over 3 months to identify characteristics of extemporaneous compounding at Primary Health Care centers. Hapsari I et al. 2018 [37].	Indonesia	1229 formulations compounded for 1200 Rx’s written	Pediatric and adult Preparations by age group 0 to 5 years—74% >5 years to <18 years—23% >18 years 2%	Paper powders 88.4% (c) Liquids (suspensions and syrups) 8% (c) Dermatological 3.6% (c)	Not provided
Cross-sectional survey of pharmacists in five districts of Yogyakarta province to assess the extent of prescription compounding in a typical month. Kristina et al. 2018 [38].	Indonesia	Survey response rate 72% (305/425) 94% (286/305) of pharmacists provided compounding services 11.55% (155/1342) Rx’s per month requesting compounding	Not specified	Paper powders 32.1% (c) Capsules 25.3% (c) Syrup 21.9% (c)	Paracetamol, Chlorpheniramine, ambroxol
Cross-sectional survey of randomly selected community and hospital pharmacies (*n* = 431) in 12 governorates assessing the prevalence of extemporaneous compounding. Alkhatib H et al. 2019 [39].	Jordan	51.7% (223/431) provided compounding services A median of 1.5 of 20 daily prescriptions contained at least 1 compounding order	Not specified	Creams 99.6% (c) Ointments 91.5% (c) Solutions 23.3% (c) Suspensions 4.5% (c) Syrup 0.9% (c)	Not provided
Cross-sectional examination of prescriptions sent to and the compounding logbook of the pharmacy department over a 4-month period in a 900-bed premier teaching hospital to determine the extent and prescribing pattern of, and labelling information provided for extemporaneous products. Yusuff KB et al. 2019 [40].	Nigeria	All (678) extemporaneous preparations were for liquid dosage form compounded for a total of 520 prescriptions involving 34 different API. 524/678 (77.2%) preparations were for pediatric patients in the Children’s Emergency Ward, while 154/678 (22.7%) were ambulatory.	Pediatric (specific age distribution not provided)	Liquid 100% (c)	Zinc gluconate, spironolactone, hydrochlorothiazide, captopril, hydroxyurea
Cross-sectional survey of hospitals in Thailand (*n* = 750). Pitchayajittipong C et al. 2021 [41].	Thailand	52.7% (395/750) response rate; 59.75% (236/395) were involved in compounding formulations	Not specified	Solid (c) Liquid (c) Semisolid (c)	Oseltamivir, isoniazid, spironolactone, furosemide, special mouthwash
Cross-sectional survey of healthcare providers’ (physicians, pharmacists, nurses) opinions on access to medicines in children collected over one month. Tiengkate P et al. 2022 [42].	Thailand	98% (218/223) response rate	Pediatric (specific age distribution not provided)	Not assessed	Omeprazole, prednisolone, sildenafil, spironolactone, aspirin
Evaluation of the type and frequency of extemporaneous compounding through an online survey administered to all community and hospital pharmacists registered with Pharmaceutical Society of Ireland. Ramtoola Z et al. 2023 [43].	Republic of Ireland	202/4361 (4.6%) pharmacists completed the survey. 170/202 prepared extemporaneous preparations 168 average number of Rx’s per month received for compounding	Pediatric and adult	Dermatological 56% (c) community pharmacies Adult and under-12 years of age oral liquid formulations 11% (c) hospital pharmacies	Not provided
Cross-sectional survey of 395 hospitals with on-site pharmaceutical production facilities to examine extemporaneous compounding practices over a 3-month period. Supapaan TS et al. 2023. [44].	Thailand	88/395 agreed to participate A total of 61 extemporaneous medicinal formulations were compounded		Oral liquids 57% (c) Semisolids 4.5% (c) Ophthalmic 38.4% (c)	Oseltamivir, isoniazid, spironolactone, furosemide, sodium bicarbonate
Survey of the International Pharmaceutical Federation (FIP) Pediatric Formulations Focus Group (PFFG). Fadda HM et al. 2024 [26].	WHO regions of the FIP PFFG Africa, Americas, Eastern Mediterranean, Europe, South-East Asia, Western Pacific	58% (736/1274) response rate 37.6% (479/1274) included in analysis	Pediatric	Oral liquids 90% (c) Capsules 44% (c) Powder papers 43% (c)	Omeprazole, captopril, spironolactone, propranolol, furosemide

c, compounded; m, manipulated; Rx’s, prescriptions.

**Table 2:** Information Resources Supporting Extemporaneous Compounding.

Country and Source	Source Type	Web Address
Children’s Hospital of Eastern Ontario, Ontario, Canada	Online Free	Pharmacy compounding formulas—CHEO
The Hospital for Sick Children, Montreal, Canada	Online Free	Pharmacy Compounding Recipes | SickKids
Chu Sainte-Justine, Montreal, Canada	Online Free	Magistrales stadnardisées au Québec—Chu Sainte-Justine
The Montreal Children’s Hospital		
Formulaire National, France	Online Free	https://ansm.sante.fr/pharmacopee/formulaire-national (accessed on 14 January 2026)
Base de datos de formulas magistrales de la SEFH, Spain	Online Free	https://gruposdetrabajo.sefh.es/farmacotecnia/formulas-magistrales (accessed on 14 January 2026)
US Pharmacist Compounding	Online Free	https://www.uspharmacist.com/topic/compounding (accessed on 14 January 2026)
Fagron Formulary	Online—certain formulations available free; others need paid membership	https://www.fagron.com/formulary (accessed on 14 January 2026)
Nationwide Children’s Hospital, US	Online Free	https://www.nationwidechildrens.org/specialties/pharmacy-services/compounding-formulas (accessed on 14 January 2026)
Deutscher Arzneimittel-Codex Neues Rezeptur-Formularium (DAC/NRF), Germany	Online registration necessary	https://dacnrf.pharmazeutische-zeitung.de/dac/nrf-wissen/rezepturenfinder/offen (accessed on 14 January 2026)

**Table 3:** Excipients with Potential Harmful Effects on Pediatric Patients [53,54].

Excipients	Harmful Effects and Recommendations	Admissible Amount for Intake
Artificial Sweeteners		
Aspartame	Headaches, seizures, neurotoxicity, hallucinations Use contraindicated in patients with phenylketonuria	40 mg/kg/day
Saccharine	Nausea, diarrhea, hives, itching, increased sensitivity to light. Limit use in pregnant women and children.	2.5 mg/kg/day
Sorbitol	Gastrointestinal disorders such as diarrhea, flatulence, abdominal pain. Hepatotoxicity. Do not use in patients with fructose intolerance. Use not recommended in hypoglycemic patients	0 to 2 years of age 5 mg/kg/day >2 years of age 140 mg/kg/day
Sucralose	Composition of gut microbiome, blood glucose, insulin and glucagon-like peptide levels altered	15 mg/kg/day
Sucrose	Dental carries. Use not recommended in children with type 1 diabetes	Not available
Preservatives		
Benzalkonium Chloride	Bronchoconstriction—use with caution in patients with asthma. Ototoxicity, hypersensitivity	Not available
Benzyl alcohol	Metabolic acidosis and respiratory depression—contraindicated in newborns and children under 3 years of age due to immature metabolism. Cerebral palsy and developmental delay. Hypersensitivity	5 mg/kg/day
Parabens (methyl, ethyl, propyl)	Cross reactions in patients allergic to acetylsalicylic acid. Avoid use in newborns and neonates to avoid hyperbilirubinemia	10 mg/kg/day
Solvents and Solubilizing Agents		
Ethyl alcohol	Hypoglycemia, acidosis, electrolyte abnormalities. CNS effects such as stupor, coma Can also be used as a preservative	6 mg/kg/dose Acceptable ethanol content in a formulation: >12 years of age = <10% *v*/*v* 6–12 years of age = <5% *v*/*v* <6 years of age = <0.5% *v*/*v*
Polyethylene Glycol	Gastrointestinal: diarrhea, flatulence, abdominal pain Nephrotoxicity Use with caution in newborns and infants	10 mg/kg/day
Propylene Glycol	CNS depression; diarrhea on oral administration due to high osmolality. Avoid in children < 4 years of age due to immature metabolism	Neonates: 1 mg/kg/day <5 years of age: 50 mg/kg/day Adults: 500 mg/kg/day
Coloring Agents		
Tartrazines, quinolines, triphenylmethane, xanthines	Hypersensitivity reactions in patients sensitive to tartrazines Azo colorants share cross-sensitivity with acetylsalicylic acid. Use in pediatric formulations not recommended	Not available

**Table 4:** United States Pharmacopeia BUDs ^1^ for Compounded Nonsterile Preparations.

Formulation Category	USP <795> (2014)	USP <795> (2023)
Aqueous—Oral	Water-containing oral formulations: 14 days (refrigerated)	Non-preserved aqueous (“a_w” ≥ 0.60): 14 days (refrigerated) [49]
Aqueous—Preserved (Oral)		Preserved aqueous (“a_w” ≥ 0.60): 35 days (controlled room temperature or refrigerated) [49]
Aqueous—Topical/Dermal/Mucosal	Water-containing topical/dermal/mucosal/oral formulations: 30 days	Managed as “aqueous” based on “a_w” and presence/absence of preservative (apply the 14- or 35-day defaults as appropriate) [49]
Nonaqueous—Oral Liquids		Nonaqueous oral liquids (“a_w” < 0.60): 90 days (controlled room temperature or refrigerated) [49]
Nonaqueous—Other Dosage Forms	Nonaqueous formulations: 6 months (180 days)	Other nonaqueous dosage forms (e.g., capsules, tablets, granules, powders) (“a_w” < 0.60): 180 days (controlled room temperature or refrigerated) [49]

^1^ BUD by type of preparation only in the absence of a USP–NF Compounded Preparation Monograph or formulation-specific stability information.

## Data Availability

No new data were created or analyzed in this study.

## Abbreviations

The following abbreviations are used in this manuscript:

APIs	Active Pharmaceutical Ingredients
ASHP	American Society of Health-System Pharmacists
BPCA	Best Pharmaceuticals for Children Act
BUDs	Beyond-Use Dates
C	Compounded
CSPs	Compounded Sterile Preparations
cGMP	current Good Manufacturing Practices
EMA	European Medicines Agency
EU	European Union
FD&C Act	Federal Food, Drug, and Cosmetic Act
FDA	Food and Drug Administration
ISO	International Standards Organization
M	Manipulated
EMLc	Model List of Essential Medicines for Children
NG	Nasogastric
NJ	Nasojejunal
NF	National Formulary
PIPs	Pediatric Investigation plans
PREA	Pediatric Research Equity Act
Rx’s	Prescriptions
3-D	Three-dimensional
US	United States of America
USP	United States Pharmacopeia
WHO	World Health Organization
